# Characterization of the Zebrafish Glycine Receptor Family Reveals Insights Into Glycine Receptor Structure Function and Stoichiometry

**DOI:** 10.3389/fnmol.2018.00286

**Published:** 2018-09-03

**Authors:** Sean Eric Low, Daishi Ito, Hiromi Hirata

**Affiliations:** Department of Chemistry and Biological Science, Aoyama Gakuin University, Sagamihara, Japan

**Keywords:** zebrafish, glycine receptor, picrotoxin, sensorimotor, hyperekplexia

## Abstract

To study characterization of zebrafish glycine receptors (zGlyRs), we assessed expression and function of five α- and two ß-subunit encoding GlyR in zebrafish. Our qPCR analysis revealed variable expression during development, while *in situ* hybridizations uncovered expression in the hindbrain and spinal cord; a finding consistent with the reported expression of GlyR subunits in these tissues from other organisms. Electrophysiological recordings using *Xenopus* oocytes revealed that all five α subunits form homomeric receptors activated by glycine, and inhibited by strychnine and picrotoxin. In contrast, ß subunits only formed functional heteromeric receptors when co-expressed with α subunits. Curiously, the second transmembranes of both ß subunits were found to lack a phenylalanine at the sixth position that is commonly associated with conferring picrotoxin resistance to heteromeric receptors. Consistent with the absence of phenylalanines at the sixth position, heteromeric zGlyRs often lacked significant picrotoxin resistance. Subsequent efforts revealed that resistance to picrotoxin in both zebrafish and human heteromeric GlyRs involves known residues within transmembrane 2, as well as previously unknown residues within transmembrane 3. We also found that a dominant mutation in human GlyRα1 that gives rise to hyperekplexia, and recessive mutations in zebrafish GlyRßb that underlie the *bandoneon* family of motor mutants, result in reduced receptor function. Lastly, through the use of a concatenated construct we demonstrate that zebrafish heteromeric receptors assemble with a stoichiometry of 3α:2ß. Collectively, our findings have furthered our knowledge regarding the assembly of heteromeric receptors, and the molecular basis of ß subunit-conferred picrotoxin resistance. These results should aid in future investigations of glycinergic signaling in zebrafish and mammals.

## Introduction

Glycine, the major inhibitory neurotransmitter in the brain stem and spinal cord, contributes to the control of motor pattern generation, the synchronization of spinal reflexes, and the processing of sensory stimuli (Lynch, 2004, 2009; Betz and Laube, 2006; Dutertre et al., 2012). Glycine exerts its effect through the opening of chloride-permeable channels termed glycine receptors (GlyRs). Structurally, GlyRs belong to the Cys-loop superfamily of receptors that in vertebrates includes chloride-permeable GABA receptors, and the cationic-permeable acetylcholine, serotonin and glutamate receptors. As a member of this family, GlyRs are comprised of five subunits, each possessing a large N-terminal extracellular domain and four transmembrane domains, the second of which lines the pore (Pribilla et al., 1992; Bormann et al., 1993; Du et al., 2015; Gielen et al., 2015; Huang et al., 2015). In addition to comprising the inter-subunit binding site for glycine, and the competitive antagonist strychnine, the N-terminal domains of all GlyR subunits contain a signal peptide sequence that promotes the translocation of mRNA-bound ribosomes to the ER whereupon translation continues. Thereafter, the signal peptide is cleaved and subunits oligomerize into pentameric receptors comprised of either all α subunits, or a mix of α and ß subunits, before being allowed to traffic to the plasma membrane. Although ß subunits lack the ability to form functional homomeric receptors, due to an absence of critical residues within several N-terminal assembly motifs (Griffon et al., 1999), ß subunits are nonetheless essential for the synaptic localization of GlyRs owing to the ability of the intracellular loop between transmembranes three and four of ß subunits (ß-loop) to bind to the postsynaptic scaffolding gephyrin (Meyer et al., 1995). This requirement of ß subunits for the synaptic localization of GlyRs has raised questions regarding the subunit stoichiometry of heteromeric GlyRs, with conflicting experimental evidence in support of either 3α:2ß or 2α:3ß (Langosch et al., 1988; Kuhse et al., 1993; Burzomato et al., 2003; Grudzinska et al., 2005; Durisic et al., 2012; Yang et al., 2012).

To date four members of the zebrafish family have been characterized in detail. The first, zebrafish GlyR (zGlyR)α1 was found to form functional homomeric receptors when expressed in *Xenopus* oocytes and mammalian cells (David-Watine et al., 1999). The second and third zGlyR subunits identified were tentatively named zGlyRα2 and zGlyRß (Imboden et al., 2001a,c), however, subsequent phylogenetic analysis and completion of the zebrafish genome necessitated their reassignments as zGlyRα4a and zGlyRßa, respectively (Imboden et al., 2001b). Curiously, the initially recovered signal peptide sequence of zGlyRα4a was found to be insufficient for the generation of glycine-evoked currents, while a chimera containing the signal peptide sequence from zGlyRα1 resulted in functional homomeric zGlyRα4a receptors (Imboden et al., 2001a). Although this finding raised the possibility that zebrafish zGlyRα4a might represent a pseudogene similar to human GlyRα4 (Simon et al., 2004; Leacock et al., 2018), a subsequent study found that an alternate variant of zGlyRα4a containing a different signal peptide sequence was capable of compensating for the loss of GlyR expression in zebrafish (Hirata et al., 2013), a finding consistent with the alternate variant being functional. Lastly, several zebrafish mutants uncovered in forward genetic screens for abnormal sensory-evoked motor behaviors have been found to arise from mutations in zGlyRßb (Granato et al., 1996; Hirata et al., 2005; Ganser et al., 2013). Mutations in zGlyRßb, collectively known as *bandoneon* mutants, result in bilateral muscle contractions in the trunk and tail due to a loss of reciprocal inhibition in the spinal cord. Similarly, mutations in human GlyRß and GlyRα1 have been shown to cause an excessive startle response disorder known as hyperekplexia (Shiang et al., 1993; Rees et al., 2002; Chung et al., 2013; James et al., 2013). This finding demonstrates the utility of using zebrafish to gain insight into human neurological disorders, and highlights the need for a better understanding of the zGlyR family.

In order to gain a more complete picture of zGlyRs we cloned and characterized each subunit. These efforts revealed that the family is comprised of five α and two ß subunits that exhibit variable expression patterns during development. All five α subunits form homomeric receptors, while ß subunits complex with α subunits at a stoichiometry of 3α:2ß to form heteromeric receptors. Subsequent analysis of mutations linked to motor impairment in zebrafish uncovered that mutated subunits often formed hypomorphic receptors. Lastly, zGlyRß subunits typically failed to confer significant picrotoxin resistance to heteromeric zGlyRα/ß receptors in accordance with the composition of amino acids in transmembrane domain 2. A closer inspection found that known residues in transmembrane domain 2, as well as previously unknown residues in transmembrane domain 3 of GlyRß subunits contribute to picrotoxin resistance in both zebrafish and human heteromeric receptors.

## Materials and Methods

### Reagents

Unless otherwise noted, all chemicals and reagents were obtained from Wako Pure Chemical Industries and Thermo Fisher Scientific, and used according to manufacturer’s guidelines.

### Animal Care and Use

Zebrafish were bred and used according to protocols set forth by the institutional animal care and use committee at Aoyama Gakuin University. Embryos reared in a 28.5°C incubator were staged using established guidelines (Kimmel et al., 1995), and are given as hours post-fertilization (hpf).

### Molecular Biology, qPCR and *in situ* Hybridization

Full-length cDNAs encoding zGlyR subunits were obtained by RT-PCR using an oligo-dT primer, SuperScript^®^ IV, and total RNA harvested from 48 hpf to 60 hpf larvae with TRIzol^®^. To each cDNA in pCS2+ a Kozak sequence of GCCGCCACC was added before the initial methionine codon to promote translation. Concatemers were constructed by substitution of zGlyRα1’s stop codon with non-redundant nucleotides encoding a 7-fold repeat of alanine-glycine-serine, followed by the post-signal peptide sequence of zGlyRßb. Site-directed mutagenesis was performed using 50 ng of donor template, 20 pmol of mutagenic primers, and 1 unit of Phusion^®^ High-Fidelity DNA Polymerase (New England Biolabs) in a final volume of 50 μl. Prior to transformation the donor template was destroyed by adding 10 units of DpnI and incubating at 37°C for 1–2 h. Capped cRNAs for expression in *Xenopus laevis* oocytes were synthesized from linearized templates using an SP6 mMessage mMachine^®^ kit (Ambion).

qPCR analysis was performed on cDNA synthesized from total RNA extracted from 50 to 100 embryos/larvae for each stage using gene-specific primers (Table 1) and KAPA FAST SYBR^™^ according to manufacturer’s guidelines. Relative expression levels were quantified using: 2∧(*Ct*(ß *actin*) − *Ct*(*zGlyR subunit*)).

**Table 1:** qPCR and riboprobe primers.

Gene	qPCR	Tm	Size (bp)	Riboprobe	Size (bp)	3′ target
zGlyRα1	F: CTCTCTTCCCAAGGTCTCG	53	171	F: AGGAGAAGGCATCTGAAGGAGGAC	489	CDS+UTR
	R: GCCTCGTCCTCCTTCAG	52		R: CAGGTCCGGATTATTCAGGAGGATA		
zGlyRα2	F: CTGTACAGCATCAGGCTGAC	54	109	F: CCACTGGCGTTCTTAATCTTCAATG	408	CDS+UTR
	R: TGGTGTATCCGAAGCTCTCC	54		R: GGTGAAATGTAACAGAGTTTGGTGAGA		
zGlyRα3	F: GCAGCTGGAGAGTTTTGGTTAC	55	166	F: GAACTGCATATCGCTGAACTCTGGT	416	UTR
	R: GCATGTGAACTTGCCTGTGTTG	55		R: TGATCTCTGCTCTTGCACTCTGCTA		
zGlyRα4a	F: GAATGTGCTTTACAGCATCAGGC	55	122	F: AGCGAAGGCAGAGAATAGAGGAAGA	433	CDS+UTR
	R: CGTTCATGGTGTAGCCAAAGC	54		R: ACTGACGGCATTTCTGGAGTCAATA		
zGlyRα4b	F: GTATAGCATCAGACTCACGCTG	55	118	F: CTCTCTGAAGCTGCTGCCATGTT	476	CDS+UTR
	R: CAGATCATTCATAGTGTAGCCAAAGC	56		R: ACAAATGTGCTCTGTGCAAAAACAA		
zGlyRßa	F: CGGCCGAATTTCAAAGGAATC	52	109	F: ATGTGCGTGTGTGTGCTTTTATGTT	438	UTR
	R: GCAGGAAGATATTCACACGATAATCCATT	57		R: TGTATGGCTCAAAAACAGCAGTTCA		
zGlyRßb	F: GTTCTCATCAGCATGAGGTTGTC	55	120	F: CATCCTGCTTCGATTCAACTCACTT	478	UTR
	R: GTCATCTGTGGTGTAACCAAAGC	55		R: TTGGTGTTCGATTCACAAGAACAGA		
ß-actin	F: ACTTTGAGCTCCTCCACACG	54	116			
	R: AGTGCGGCAATTTCATCATC	50				

*(F) forward and (R) reverse primer, (Tm) melting temperature, (CDS) coding sequence, (UTR) untranslated region*.

Templates for zGlyR riboprobes were isolated using primers raised against either divergent coding sequence or 3’ untranslated regions (Table 1). DIG-conjugated riboprobes were synthesized and used according to established procedures (Low et al., 2011). Images were captured using a ProgRes^®^ CF Scan camera and associated software (Jenoptik), where after auto contrast in Photoshop CS3 (Adobe) was employed.

### Electrophysiology

Oocytes were injected with five femtomoles of cRNA using a Nanoject II (Drummond Scientific), where after oocytes were incubated in Barth’s solution (in mM: 88 NaCl, 1 KCl, 2.4 NaHCO_3_, 0.33 Ca(NO_3_)_2_, 0.41 CaCl_2_, 0.82 MgSO_4_, 10 HEPES at pH 7.5 with NaOH, supplemented with gentamicin at 50 μg/ml, and penicillin/streptomycin at 100 units/ml) at 17°C for 24–72 h prior to recording. To determine the distribution of heteromeric receptor assembly we used the following equation: Probability of receptor subtype = (*K*) * *p*^α^ * (1 – *p*)^ß^, where “p” is the proportion of cRNA encoding a subunit, “α” and “ß” are the numbers of α and ß subunits in a receptor subtype, and “K” is the number of ways a receptor subtype can form during receptor assembly: 1 for 5α:0ß and 0α:5ß, 5 for 4α:1ß and 1α:4ß, and 10 for 3α:2ß and 2α:3ß. Please note that functional homomeric ß receptors (0α:5ß) are not made when determining the percentage of receptor subtypes. Oocyte recording solution (in mM: 90 NaCl, 1 KCl, 2 CaCl_2_, 1 MgCl_2_, 10 HEPES at pH 7.5 with NaOH) and up to seven other solutions were applied to oocytes using a BPS-8 solution switcher (ALA Scientific). Borosilicate electrodes had resistances of ~0.5 MΩ when filed with 3 M KCl. Two-electrode voltage-clamp recordings were made from oocytes held at −50 mV using pClamp^™^ 10.2 to control a GeneClamp^®^ 500B amplifier via a Digidata^®^ 1440A digitizer (Molecular Devices). Signals were low-pass filtered at 10 Hz, and sampled at 100 Hz. Recordings were analyzed using Clampfit 10.7 (Axon Instruments) and SigmaPlot 11.0 (Systat Software, Inc.). Statistical significance was assessed using a Mann-Whitney U test assuming equal variance at *p* < 0.05.

### Imaging

HEK293T cells (5 × 10^5^) were transfected with 2 μg of expression vectors using the Lipofectamine2000 method according to the manufactures’ protocol. Frame averaged (6×) optical slices (x/y; 4096/4096) of live HEK293T cells were captured at 400 Hz using a 40× objective (HCX APO L, NA 0.8, water) and a TCS SP5 laser-scanning confocal microscope (Leica) with a pinhole diameter of 1 Airy. Gain settings that were below saturation for venus-tagged zGlyRα1 transfected cells were subsequently used to acquire fluorescent images of venus-tagged zGlyRα1^R271Q^ transfected cells. Images were prepared using Fiji (Schindelin et al., 2012) and Adobe Photoshop CS3 wherein the Blur filter was used.

## Results

### Zebrafish Glycine Receptor Subunits Share Structural Similarities With Human Subunits

**Figure 1:**
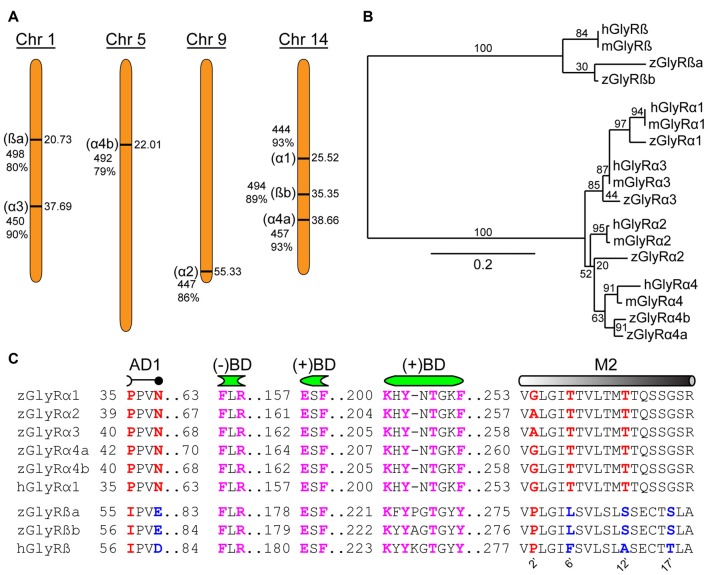
Zebrafish family of glycine receptors (GlyR). **(A)** Chromosomal (Chr) arrangement of zebrafish GlyR (zGlyR) subunits. Location (in Mbp) are on the right, while name of subunit, length and sequence similarity to human ortholog are given to the left. **(B)** Phylogenetic analysis of zGlyR subunits. Horizontal scale bar at the bottom reflects amount of genetic change, all other numbers are bootstraps. (mGlyR and hGlyR) mouse and human GlyR subunit. **(C)** Sequence alignments of zGlyRα subunits to hGlyRα1, and zGlyRß subunits to hGlyRß. Red letters indicate residues conserved among α and ß subunits from zebrafish and human. Blue letters indicate residues conserved between zGlyRß paralogs which differ from hGlyRß. Magenta letters indicate residues conserved between α and ß subunits from zebrafish and human. AD1: assembly domain one, (−)BD and (+)BD: negative and positive faces of the inter-subunit binding sites for glycine and strychnine, M2: transmembrane domain 2. Numbered residues (‘) indicate position with M2.

An assessment of the GRCz11 genome assembly indicated the presence of seven putative open reading frames encoding zGlyR subunits (Figure 1A). Subsequent cloning and sequence analysis revealed that zebrafish possess single orthologs of α1, α2 and α3, and two orthologs of α4 (α4a and α4b) and ß (ßa and ßb). Phylogenetic analysis revealed that α3 subfamily is close to α1 subfamily, and α4 close to α2 (Figure 1B). The duplicated α4 and ß subunits are herein referred to as paralogs in accordance with established guidelines (Wood, 1998).

A closer inspection of the amino acid content of each zGlyR subunit revealed the following notable features. Residues thought to form the positive (+) and negative (−) portions of the inter-subunit binding sites for glycine and strychnine (Vandenberg et al., 1992; Grudzinska et al., 2005), as well as the receptor’s preference for glycine over closely related molecules such as ß-alanine, GABA and D-serine (Schmieden et al., 1993), are conserved (Figure 1C). In addition, the proline and asparagine residues in assembly domain one that are essential for oligomerization of GlyR subunits in the ER, a necessary step in the translocation of assembled GlyRs out of the ER (Griffon et al., 1999), are present in all five zGlyRα subunits, but are absent in both zGlyRß subunits. Therefore, like their mammalian orthologs, zGlyRß subunits are unlikely to form functional homomeric receptors.

In a previous study, the second transmembrane domains (M2s) of GlyR subunits have been shown to be related to function (Shan et al., 2001). The second residue (2’) of the M2 within GlyRα subunits is occupied by a glycine or alanine, as opposed to a proline typical of ß subunits (Figure 1C). While a glycine or alanine at the 2’ position in the human GlyRα1 subunit (hGlyRα1^G254A^) was found to have no effect on the excitatory amount of glycine required to half-maximally activate receptors (EC_50_), substitution to the “ß-like” proline (hGlyRα1^G254P^) resulted in a ~6 fold increase in the EC_50_ for glycine (Shan et al., 2001). The other intriguing feature of M2 was the presence of a leucine at the 6’ position in zGlyRß subunits, which is normally occupied by a phenylalanine in ß subunits from the other vertebrate species (Hirata et al., 2009). The phenylalanine at the 6’ position has been shown to be both necessary for ß subunit-conferred picrotoxin resistance to heteromeric hGlyRα1/ß receptors, and sufficient to confer resistance to homomeric hGlyRα1 receptors (Shan et al., 2001). Taken together, homomeric zGlyRα receptors would be expected to exhibit moderate EC_50s_ for glycine, while heteromeric zGlyRα1/ß receptors might manifest as receptors with reduced picrotoxin resistance.

### zGlyR Subunits Exhibit Variable Expression in the Nervous System During Development

**Figure 2:**
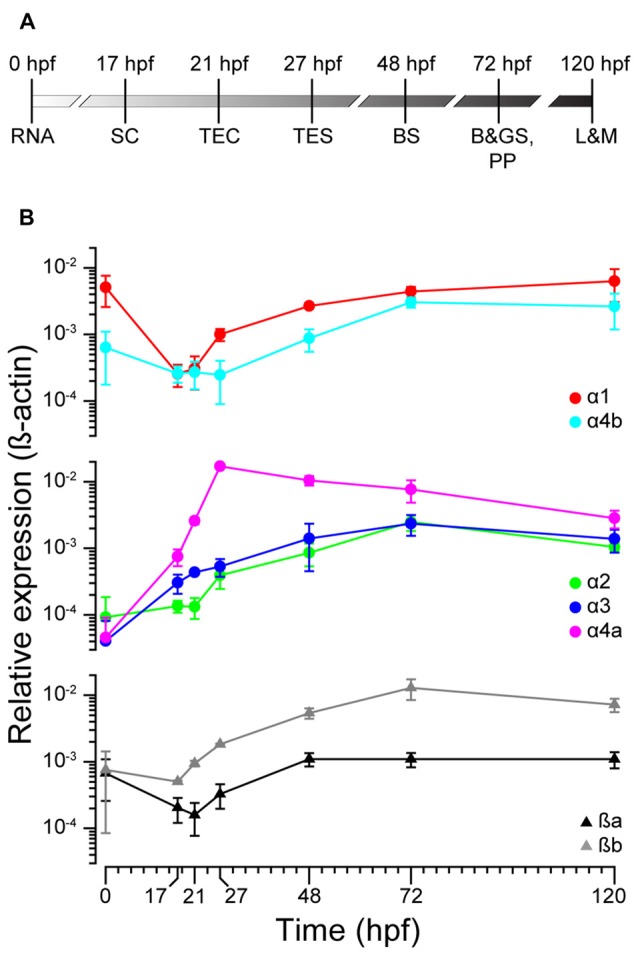
zGlyR subunits show variable expression during development. **(A)** Timeline highlighting the developmental stages examined. RNA, inheritance of maternal RNA; SC, spontaneous coiling; TEC, touch-evoked coiling; TES, touch-evoked swimming; BS, burst swimming; B&GS, PP: beat and glide swimming, prone position; L&M, learning and memory. **(B)** Expression of zGlyR subunits over time determined by qPCR. Each subunit’s expression level was normalized against b-actin. Values represent the average ± SEM of nine samples from three different mating pairs.

To ascertain the expression profiles of zGlyR subunits during development, we performed qPCR at time points that coincided with the following (Figure 2A): the inheritance of maternal RNA (0 hpf), the onset of spontaneous motor activity (17 hpf), and the emergence of touch-evoked contractions (21 hpf) and swimming (27 hpf), both of which involve glycine-dependent reciprocal inhibition between the bilateral halves of the spinal cord (Saint-Amant and Drapeau, 2000; Hirata et al., 2005). In addition, we examined commonly employed stages of development including when embryos exhibit “burst” swimming (48 hpf), larvae adopt the prone position and convert to “beat and glide” swimming (72 hpf), and when larvae possess many adult-like abilities such as learning and memory (120 hpf). We found that transcripts encoding zGlyRα1 and zGlyRα4b were dominant among the zGlyRα subunits in inherited maternal RNA (Figure 2B), while RNA encoding zGlyRßa and zGlyRßb subunits were equally represented. Thereafter, RNA encoding these four subunits all displayed a modest reduction at the onset of spontaneous motor activity, while at the same time RNA encoding zGlyRα2 remained constant, and RNA encoding zGlyRα3 and zGlyRα4a increased. The cumulative effect resulted in a decrease in the extent of RNA variation from 125-fold at 0 hpf (zGlyRα1 vs. zGlyRα3) to 6-fold at 17 hpf (zGlyRα4a vs. zGlyRα2). Thereafter, all RNAs displayed either an immediate or delayed increase in representation until reaching an apparent steady state between 72 and 120 hpf with a 7-fold extent of RNA variation (zGlyRα2 vs. zGlyRßb).

**Figure 3:**
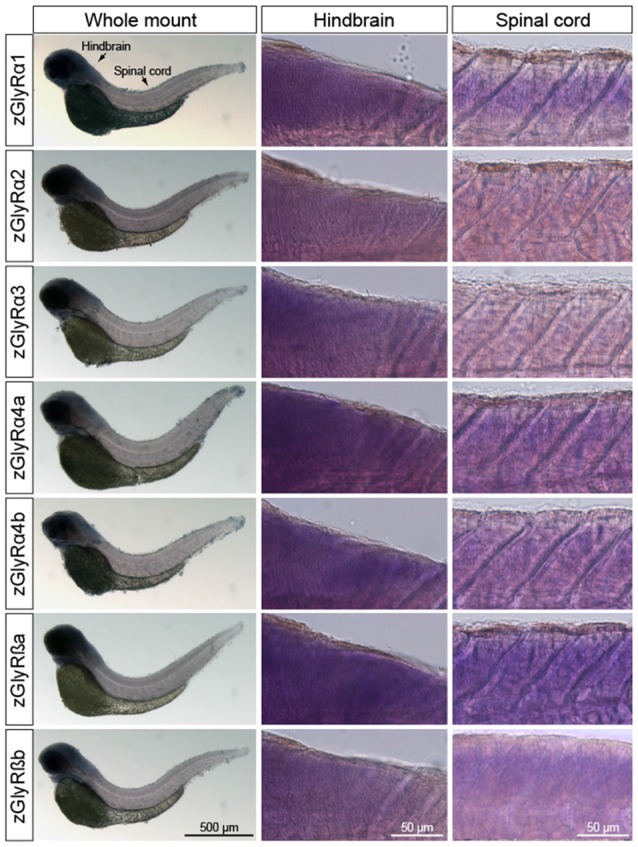
Whole-mount *in situ* hybridizations of zGlyR subunits from 48 hours post-fertilization (hpf) larvae. Individual subunits are indicated to the left.

In parallel, we also performed whole-mount *in situ* hybridizations in an attempt to determine which tissues expressed zGlyR subunits. To this end we utilized 48 and 120 hpf larvae as all zGlyR subunits were expressed at these time points (Figure 2B). We found that zGlyRα4a transcripts were present in eye, consistent with a previous report (Hensley et al., 2011). Whereas all subunits were expressed in the hindbrain of 48 hpf larvae (Figure 3), transcripts for zGlyRα1, zGlyRα2, zGlyRα4a, zGlyRα4b, zGlyRßa and zGlyRßb were detected in the spinal cord. Likewise, at 120 hpf all transcripts were again detected in the hindbrain (Supplementary Figure S1), while zGlyRα1 and zGlyRα3 were detected in the spinal cord. These findings were consistent with the observed expression pattern of mammalian GlyRs (Zarbin et al., 1981; Probst et al., 1986).

### zGlyRα Subunits Form Homomeric and Heteromeric Receptors With zGlyRß Subunits

**Figure 4:**
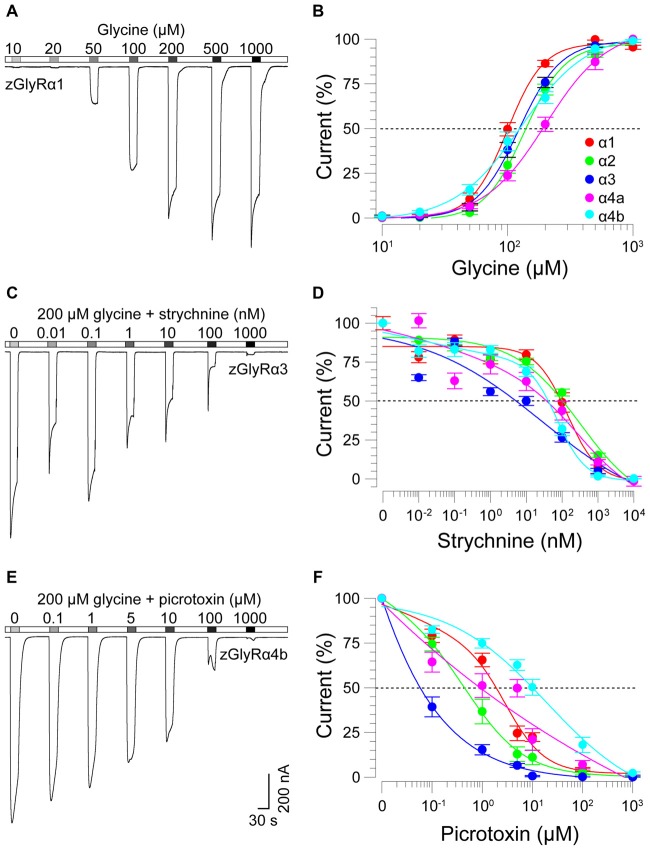
zGlyRα subunits form functional homomeric receptors that are activated by glycine, and inhibited by strychnine and picrotoxin. **(A)** Two-electrode voltage-clamp recording from an oocyte injected with five femtomoles of zGlyRα1 cRNA exposed to serial application of glycine of increasing amount. Ten oocytes were used for each assay. **(B)** Cumulative dose-response relationship of glycine-evoked currents. The amplitude of each glycine-evoked response was normalized to the maximally-evoked current for each oocyte (*n* = 10). Values here and elsewhere represent the average ± SEM. Dashed line denotes EC_50_. **(C,E)** Recordings from oocytes exposed to glycine and increasing amounts of strychnine or picrotoxin, respectively (*n* = 10). **(D,F)** Cumulative dose-response relationships of strychnine and picrotoxin-blocked currents (*n* = 10). Extent of blockade was normalized to the current amplitude evoked by 200 μM glycine in each oocyte. Dashed lines denote IC_50s_.

To determine the functionality of zGlyR subunits, we first made two-electrode voltage-clamp recordings from *Xenopus* oocytes injected with cRNA encoding a single subunit. This approach revealed that each zGlyRα subunit, including the alternate variant of zGlyRα4a (Hirata et al., 2013), formed functional homomeric receptors that were activated by micromolar amounts of glycine (Figures 4A,B), and inhibited by nanomolar amounts of strychnine and micromolar amounts of picrotoxin (Figures 4C–F; Table 2). In contrast, both zGlyRß subunits failed to yield currents above background (not shown), a finding in agreement with the absence of necessary residues within assembly domain one of zGlyRß subunits (Griffon et al., 1999).

**Table 2:** Summary of homomeric and heteromeric zGlyR properties.

	Glycine		Strychnine		Picrotoxin	
zGlyR	EC_50_ [μM]	Hill coeff.	IC_50_ [nM]	Hill coeff.	IC_50_ [μM]	Hill coeff.
α1	102 ± 5.8**	3.3 ± 0.2	136 ± 22	1.1 ± 0.2	2.7 ± 0.7	0.8 ± 0.1
α1/ßa	186 ± 10***	2.2 ± 0.2**	12 ± 4.0***	0.6 ± 0.0	5.1 ± 2.0	1.0 ± 0.1
α1/ßb	194 ± 18***	2.1 ± 0.2**	74 ± 17*	0.7 ± 0.1	23 ± 11*	0.8 ± 0.1
α2	138 ± 10	3.0 ± 0.1	212 ± 37	0.7 ± 0.1	0.9 ± 0.4	0.8 ± 0.0
α2/ßa	307 ± 30***	2.3 ± 0.3*	292 ± 43	1.0 ± 0.1**	23 ± 5.1***	0.8 ± 0.1
α2/ßb	343 ± 64**	1.7 ± 0.2***	121 ± 16*	0.8 ± 0.1	24 ± 7.8**	0.8 ± 0.1
α3	125 ± 7.9	2.8 ± 0.1	21 ± 8.0	0.4 ± 0.1	0.1 ± 0.0	0.6 ± 0.1
α3/ßa	521 ± 52***	1.7 ± 0.1***	83 ± 16**	0.6 ± 0.1**	4.1 ± 0.8***	1.0 ± 0.2*
α3/ßb	260 ± 20***	1.9 ± 0.1***	41 ± 8.1	0.6 ± 0.0***	10 ± 1.0***	1.3 ± 0.2**
α4a	200 ± 16	2.2 ± 0.2	55 ± 12	0.9 ± 0.2	5.5 ± 3.8	0.3 ± 0.1
α4a/ßa	458 ± 82**	2.2 ± 0.2	43 ± 22	0.9 ± 0.1	1.6 ± 0.3	0.6 ± 0.1*
α4a/ßb	404 ± 24***	2.2 ± 0.1	18 ± 2.6**	0.7 ± 0.1	1.5 ± 0.6	0.7 ± 0.1*
α4b	134 ± 15***	1.8 ± 0.1	52 ± 11	0.9 ± 0.1	31 ± 13	0.6 ± 0.1
α4b/ßa	236 ± 17***	1.9 ± 0.1	81 ± 16	1.3 ± 0.1	8.5 ± 3.3	0.5 ± 0.0
α4b/ßb	221 ± 15***	1.9 ± 0.1	21 ± 3.1*	1.1 ± 0.1	20 ± 5.7	0.6 ± 0.1

Values represent the average ± SEM from 10 oocytes each. Where indicated *p < 0.05, **p < 0.01, and ***p < 0.001 reflect difference between heteromeric and homomeric receptors.

**Figure 5:**
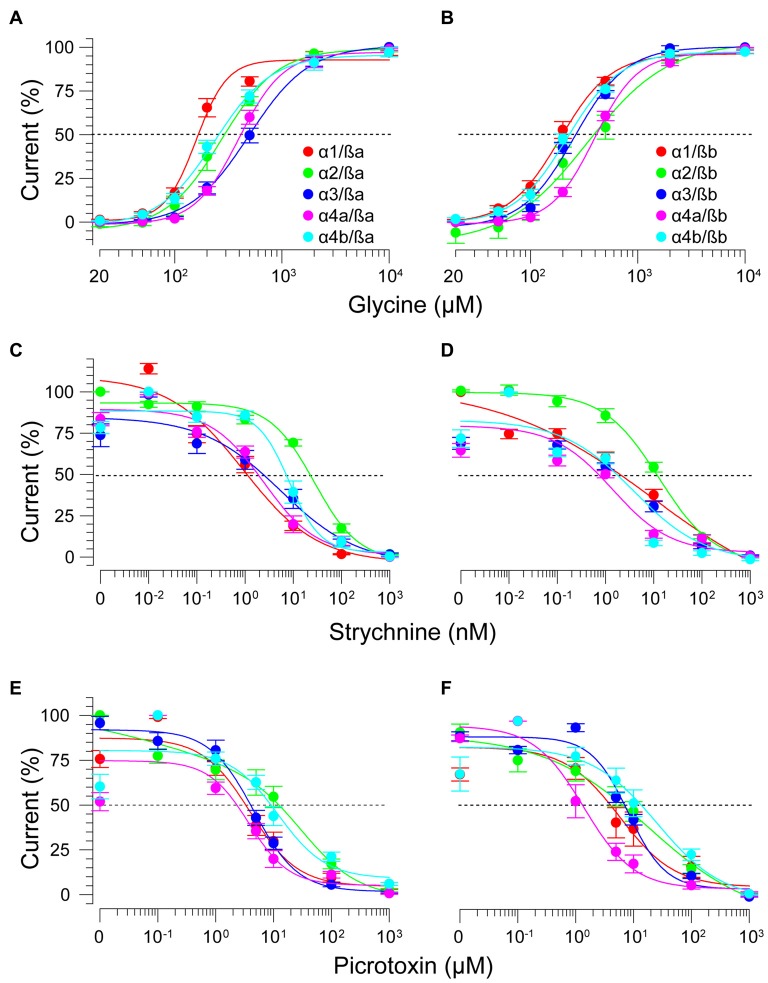
zGlyRß subunits form functional heteromeric receptors with zGlyRα subunits. Cumulative dose-response relationships of glycine-evoked currents from oocytes co-injected with 2.5 femtomoles of zGlyRα cRNA and 2.5 femtomoles of either zGlyRßa **(A)** or zGlyRßb **(B)** cRNA. Ten oocytes were used for each assay. **(C–F)** Cumulative dose-response relationships of strychnine and picrotoxin-blocked currents from heteromeric zGlyRs. Ten oocytes were used for each assay.

As ß subunits from other organisms complex with α subunits to form heteromeric receptors (Pfeiffer et al., 1982; Grenningloh et al., 1990; Pribilla et al., 1992), we next recorded from oocytes co-expressing each zGlyRα subunit and either zGlyRßa or zGlyRßb. We found that the co-injection of zGlyRß cRNAs consistently led to a rightward shift in the EC_50_ for glycine when compared to oocytes expressing each zGlyRα subunit alone (Figures 5A,B; Table 2); a result consistent with the formation of heteromeric GlyRs in oocytes (Kuhse et al., 1993; Langosch et al., 1994; Grudzinska et al., 2005). In contrast, the co-expression of zGlyRß subunits with zGlyRα subunits either had no effect on strychnine’s half-maximal inhibitory amount (IC_50_), or yielded mixed results. For example, both zGlyRß subunits reduced strychnine’s IC_50_ when co-expressed with zGlyRα1, while zGlyRßb reduced the IC_50_ for zGlyRα2, zGlyRα4a and zGlyRα4b (Figures 5C,D; Table 2). By comparison, zGlyRßa was found to increase strychnine’s IC_50_ when co-expressed with zGlyRα3.

Lastly, we explored the sensitivity of heteromeric zGlyRs to the pore-blocking antagonist picrotoxin. We found that oocytes expressing heteromeric zGlyRα1/ßa receptors were as sensitive to picrotoxin as those expressing homomeric zGlyRα1 receptors (Figures 5E,F). Likewise, heteromeric receptors comprised of zGlyRα4a or zGlyRα4b and either zGlyRß paralog were also indistinguishable from their homomeric zGlyRα counterparts (Table 2). In contrast, zGlyRßb rendered heteromeric zGlyRα1/ßb receptors ~8.5 fold more resistant to picrotoxin, while heteromeric receptors comprised of zGlyRßa or zGlyRßb and either zGlyRα2 or zGlyRα3 were between 25 and 100 fold more resistant. While the basis for the varying effects of zGlyRß paralogs on a receptor’s sensitivity to strychnine and picrotoxin is currently unclear, these results are nonetheless consistent with the formation of functional heteromeric zGlyRs.

### Residues Within Transmembrane Domains Two and Three of Zebrafish and Human GlyRß Subunits Contribute to Picrotoxin Resistance

**Figure 6:**
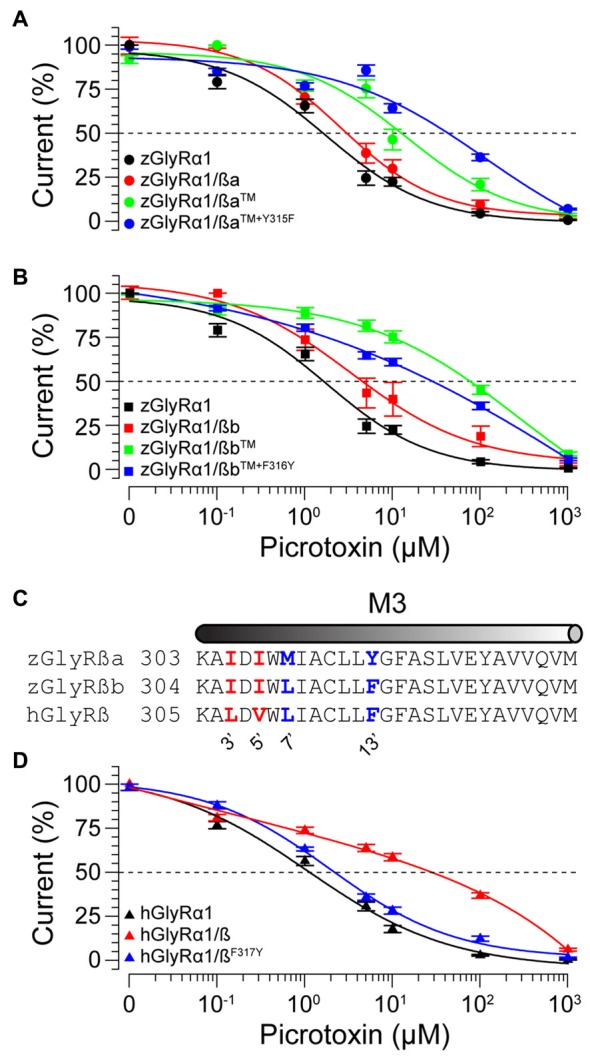
Residues within M2 and M3 of zebrafish and human ß subunits contribute to picrotoxin resistance. Cumulative effect of amino acid substitutions on picrotoxin-mediated blockade of zGlyRßa **(A)**, zGlyRßb **(B)**, and hGlyRß containing receptors **(D)**. TM: triple mutation of residues in M2 of zGlyRß subunits; ßa: L280F+S286A+S291T, ßb: L281F+S287A+S292T. Ten oocytes were used for each assay.** (C)** Sequence alignments of M3 from hGlyRß and zGlyRß subunits. Red and blue letters indicate residues that are conserved between zGlyRß subunits, and zGlyRßb and hGlyRß, respectively.

To examine the molecular basis of picrotoxin sensitivity in heteromeric zGlyRs, we chose to focus on zGlyRα1/ß receptors as previous research had identified essential residues within M2 of the human ß subunit (hGlyRß) essential for picrotoxin resistance in heteromeric hGlyRα1/ß receptors (Pribilla et al., 1992; Shan et al., 2001). A sequence comparison between the M2s of zGlyRß subunits and hGlyRß uncovered that zGlyRß subunits differ from hGlyRß at the 6’, 12’, and 17’ position (Figure 1C). Given that the conversion of the 6’ position in hGlyRß from phenylalanine to threonine abolishes ß-subunit conferred picrotoxin resistance in heteromeric receptors, and that conversion of the same position in hGlyRα1 from glycine to phenylalanine confers picrotoxin resistance to homomeric receptors (Shan et al., 2001), we first substituted the leucines at the 6’ position in both zGlyRß paralogs with phenylalanines (zGlyRßa^L280F^, zGlyRßb^L281F^). We found that the resistance of zGlyRα1/ßa^L280F^ and zGlyRα1/ßb^L281F^ receptors to picrotoxin was indistinguishable from wild-type zGlyRα1/ß receptors (Table 3), indicating that the presence of an aromatic phenylalanine at the 6’ position alone was insufficient to significantly increase picrotoxin resistance. We therefore, next made individual and combined substitutions of all divergent residues within M2, which revealed that conversion of all three residues (TM: triple mutant) induced an ~8-fold increase in picrotoxin resistance in heteromeric zGlyRα1/ßa^™^ receptors (Figure 6A). However, conversion of the same residues in heteromeric zGlyRα1/ßb^™^ receptors increased picrotoxin resistance by ~42 fold (Figure 6B); a finding which indicates that previously unidentified residues outside of M2 also contribute to picrotoxin resistance.

**Table 3:** Summary of modified ß subunits effects on picrotoxin and glycine sensitivity.

zGlyR	ß subunit	IC_50_ [μM]	Hill coeff.	EC_50_ [μM]	Hill coeff.
α1	none	2.4 ± 0.8	0.9 ± 0.1	108 ± 8.8	3.2 ± 0.1
α1/ßa	wild type	5.0 ± 1.3	1.1 ± 0.1	191 ± 11	2.1 ± 0.2
	L280F	6.3 ± 1.8	0.4 ± 0.1***		
	S286A	1.7 ± 0.4*	0.6 ± 0.0***		
	S291T	2.3 ± 0.4*	0.7 ± 0.1**		
	L280F+S286A	4.6 ± 1.8	0.9 ± 0.2		
	L280F+S291T	8.7 ± 2.5	0.6 ± 0.1**		
	S286A+S291T	6.2 ± 1.4	0.7 ± 0.0**		
	L280F+S286A+S291T	22 ± 6.0*	1.0 ± 0.2		
	L280F+S286A+S291T+M309L	10 ± 2.0	0.4 ± 0.1***		
	L280F+S286A+S291T+Y315F	44 ± 6.7***	0.4 ± 0.1***	220 ± 17	2.7 ± 0.3
	L280F+S286A+S291T+M309L+Y315F	26 ± 5.9**	0.4 ± 0.2**		
α1/ßb	wild type	20 ± 16	0.7 ± 0.2	212 ± 22	2.2 ± 0.2
	L281F	16 ± 6.2	0.5 ± 0.2		
	S287A	4.0 ± 0.7	0.8 ± 0.1		
	S292T	6.8 ± 1.9	0.5 ± 0.0		
	L281F+S287A	23 ± 6.0	0.6 ± 0.7		
	L281F+S292T	29 ± 6.7	0.3 ± 0.0**		
	S287A+S292T	3.3 ± 1.2	0.4 ± 0.0*		
	L281F+S287A+S292T	113 ± 12***	0.7 ± 0.1	259 ± 17	2.9 ± 0.2
	L281F+S287A+S292T+F316Y	40 ± 6.8	0.3 ± 0.0*	153 ± 18	3.5 ± 0.6
hGlyR					
α1	none	1.6 ± 0.3	0.6 ± 0.0	178 ± 9.2	3.2 ± 0.1
α1/ß	wild type	31 ± 5.0	0.1 ± 0.0	218 ± 11	2.2 ± 0.2
	F317Y	1.9 ± 0.1***	0.6 ± 0.1***	167 ± 18*	2.4 ± 0.3

Values represent the average ± SEM from 10 oocytes each. *p < 0.05, **p < 0.01, and ***p < 0.001 reflect difference between wild type and modified heteromeric receptors.

A further comparison of nearby residues revealed that zGlyRßa differs from zGlyRßb and hGlyRß subunits at the 7’ and 12’ positions within transmembrane domain 3 (M3; Figure 6C). Subsequent conversion of methionine to leucine at the 7’ position rendered zGlyRα1/ßa^TM+M309L^ receptors *less* resistant to picrotoxin (Table 3), while conversion of tyrosine at the 12’ position to phenylalanine yielded zGlyRα1/ßa^TM+Y315F^ receptors that were ~16 fold more resistant (Figure 6A). To determine whether the analogous residue within hGlyRß also contributes to picrotoxin resistance we engineered a hGlyRß^F317Y^ variant, which when co-expressed with hGlyRα1 yielded heteromeric receptors devoid of ß subunit-conferred picrotoxin resistance (Figure 6D), although we cannot completely exclude the possibility that hGlyRb-F317Y subunit was not incorporated into functional GlyR heteromers. Taken together, residues within M2 and M3 of both zebrafish and human ß subunits likely contribute to picrotoxin resistance.

### Mutations in zGlyRßb and zGlyRα1 Result in Hypomorphic Receptors

**Figure 7:**
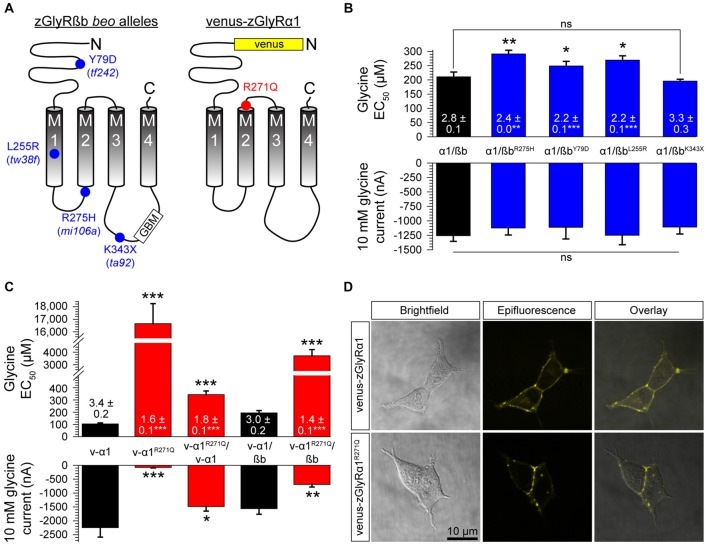
Mutations in zGlyRßb and zGlyRα1 result in hypomorphic receptors. **(A)** Location of mutations in zGlyRßb that give rise to *bandoneon* (*beo*) mutants, and analogous position in hGlyRα1 of mutations that cause hyperekplexia. (GBM) Gephyrin-binding motif. **(B)** Cumulative effects of *bandoneon* mutations on heteromeric receptors’ EC_50_ for glycine and average 10 mM glycine-evoked currents. Of note, oocytes were injected with 1 femtomole of zGlyRα1 cRNA and four femtomoles of zGlyRßb cRNAs. Ten oocytes were used for each assay. Numbers inside bar here and in **(C)** represent Hill coefficient, and *p < 0.05, **p < 0.01, and ***p < 0.001. **(C)** Effect of dominant-negative R271Q mutation on both homomeric and heteromeric receptors’ EC_50_ for glycine and average 10 mM glycine-evoked currents. Each oocyte was injected with 2.5 femtomoles of venus-tagged zGlyRα1 cRNAs and 2.5 femtomoles of zGlyRßb cRNA. Ten oocytes were used for each assay.** (D)** Confocal images of HEK293T cells expressing venus-tagged zGlyRα1 subunits.

Currently, seven alleles of the zebrafish mutant *bandoneon* exist (Granato et al., 1996; Hirata et al., 2005), which have been shown to arise from the following: three nonsense mutations that truncate zGlyRßb prior to the first transmembrane domain (not indicated), three missense mutations of unknown consequence (Figure 7A), and one adult-viable nonsense mutation that truncates zGlyRßb in the ß-loop prior to the gephyrin-binding motif and the fourth transmembrane domain (Hirata et al., 2005; Ganser et al., 2013). In an attempt to elucidate the functional consequence of the three lethal missense mutations, and lone viable nonsense mutation, we co-expressed zGlyRßb subunits bearing each mutation with zGlyRα1 in oocytes. To facilitate the formation of heteromeric receptors we co-injected oocytes with zGlyRα1 and zGlyRßb cRNAs at a ratio of 1:4. Assuming an equal probability of subunit selection during receptor assembly, a 1:4 ratio predicts that 99.9% of the GlyRs formed will be heteromeric receptors (see “Materials and Method” section). We found that the four mutations in zGlyRßb result in functional heteromeric receptors (Figure 7B). A closer inspection revealed that three of the four mutated receptors exhibited elevated EC_50_ for glycine, despite displaying similar current amplitudes in response to 10 mM glycine. The fourth mutated receptor, zGlyRα1/ßb^K343X^ was indistinguishable from wild-type heteromeric receptors, indicating that the mutant phenotype is not apparently caused by a defect in ligand binding or Cl^−^ conductance.

In parallel to the use of *bandoneon* mutants, two additional studies have sought to investigate the contribution of glycinergic signaling in zebrafish through the use of mutated zGlyRα subunits (Ganser et al., 2013; Leacock et al., 2018). In brief, a dominant form of hyperekplexia in humans is caused by mutations in hGlyRα1 that convert the arginine immediately following M2 to either leucine or glutamine (Shiang et al., 1993). Subsequent investigations revealed that the hGlyRα1^R271Q^ mutation increases EC_50_ for glycine and results in severely diminished glycine-evoked currents owing to a 84% decrease in the single channel conductance of homomeric hGlyRα1^R271Q^ receptors, and between a ~150–180-fold reduction in the sensitivity of homomeric and heteromeric receptors to glycine, respectively (Langosch et al., 1994). As this arginine residue is conserved among GlyRα subunits from different species (Hirata et al., 2009), substitution of the analogous residue in zGlyRα subunits is thought to exert a similar effect. To examine this possibility, we made recordings from oocytes injected with cRNA encoding venus-tagged zGlyRα1 bearing an arginine 271 to glutamine substitution (Figure 7A; zGlyRα1^R271Q^). We found that the zGlyRα1^R271Q^ mutation resulted in 96% reduction in glycine-evoked currents (Figure 7C), and a ~170-fold shift in the glycine EC_50_. When co-expressed with wild-type venus-tagged zGlyRα1, we observed a 3.4-fold increase in the EC_50_ for glycine, and a 33% reduction in glycine-evoked currents. Finally, co-expression of zGlyRα1^R271Q^ with zGlyRßb resulted in a ~19-fold increase in the EC_50_ for glycine, and a 56% reduction in glycine-evoked currents. Considering that a 15% increase in the EC_50_ for glycine is sufficient to impair motor activity (zGlyRß^Y79D^; Figure 7B) suggests that the ectopic expression of zGlyRα1^R271Q^ is likely to be an effective tool for disrupting glycinergic transmission *in vivo*.

Finally, to determine whether zGlyRα1^R271Q^ subunits traffic to the membrane like their human counterpart (Langosch et al., 1993), we examined the subcellular localization of venus-tagged zGlyRα1 subunits in HEK293T cells. This approach revealed that zGlyRα1^R271Q^ subunits were distributed similar to wild-type zGlyRα1 subunits, and in a manner consistent with membrane localization (Figure 7D).

### Heteromeric zGlyRs Assemble With a Subunit Stoichiometry of 3α:2ß

**Figure 8:**
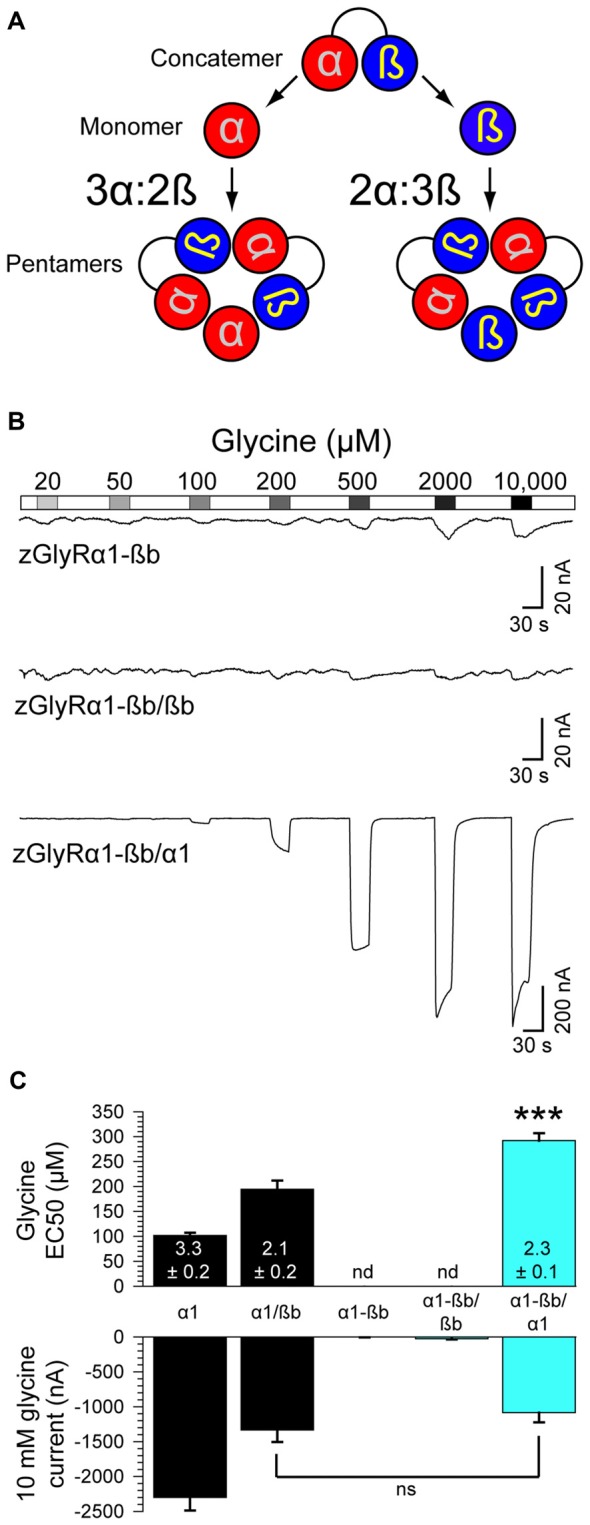
Heteromeric zGlyRs assemble with a stoichiometry of 3α:2ß. **(A)** Schematic detailing the assembly of pentameric zGlyRs from concatemers and either monomeric zGlyRα1 or zGlyRßb. **(B)** Two-electrode voltage clamp recordings from oocytes injected with cRNA encoding concatemers alone, or with monomeric zGlyRα1 or zGlyRßb cRNA. Note that the current scale bars on the top and middle traces differ from the bottom trace.** (C)** Average glycine EC_50_ and responses to 10 mM glycine (*n* = 10). ***p< 0.001.

Densiometric measurements of endogenous GlyRs purified from the spinal cords of rats using strychnine-based affinity chromatography uncovered α to ß subunit ratios most consistent with heteromeric receptors comprised of 3α:2ß (Langosch et al., 1988). However, this technique might have led to an over estimation in the number of α subunits per heteromeric receptor given that a strychnine-based approach would also isolate pentameric GlyRs composed of only α subunits. Consistent with this possibility, subsequent studies employing a concatenated hGlyRα1-ß construct and metabolic labeling of monomeric subunits in *Xenopus* oocytes (Grudzinska et al., 2005), and atomic force microscopy measurements of antibody-labeled HEK293 cell extracts (Yang et al., 2012), have suggested an invariant heteromeric stoichiometry of 2α:3ß. To explore the subunit assembly of heteromeric zGlyRs we engineered a zGlyRα1-ßb concatenated construct and expressed it with either monomeric zGlyRα1 or zGlyRßb subunits (Figure 8A).

As a control, we first determined whether the concatenated construct alone was sufficient to generate functional channels. To this end we made recordings from oocytes injected with only cRNA encoding the zGlyRα1-ßb concatenated construct. We found that oocytes displayed minimal currents in response to 10 mM glycine (3.6 ± 1.5 nA; Figure 8B), indicating a general absence of functional pseudo pentameric receptors featuring an outwardly facing sixth subunit. We therefore, next made recordings from oocytes co-injected with cRNA encoding the zGlyRα1-ßb concatemer and either monomeric zGlyRα1 or monomeric zGlyRßb. We found that oocytes co-injected with concatenated zGlyRα1-ßb and monomeric zGlyRßb cRNAs again displayed minimal glycine-evoked currents (26.4 ± 10.4 nA), indicating that 2α:3ß heteromeric receptors are not readily made. In contrast, oocytes co-injected with concatenated zGlyRα1-ßb and monomeric zGlyRα1 cRNAs exhibited glycine-evoked responses reminiscent of currents obtained from oocytes co-injected with monomeric zGlyRα1 and monomeric zGlyRßb cRNAs (Figure 8C). Hence, heteromeric zGlyRs assemble with a stoichiometry of 3α:2ß.

## Discussion

Zebrafish have proven to be a useful model organism for studies related to glycinergic neurotransmission. For instance, mutagenesis screens have uncovered mutations in the zGlyRßb that result in bilateral contractions of the trunk and tail owing to a loss of reciprocal inhibition in the spinal cord (Hirata et al., 2005). The zebrafish *bandoneon* phenotype mirrors the human neurological disorder hyperekplexia which is likewise caused by mutations in hGlyRß and hGlyRα1. This finding that simultaneously demonstrated the utility of using zebrafish to gain insight into human disorders, and highlighted the need for a better understanding of the zGlyR family. Here, we detail fundamental characteristics of all seven zGlyR subunits.

### The Potential Contribution of Maternally-Inherited RNA to Behavior and Neurogenesis

The earliest behavior exhibited by zebrafish is spontaneous coiling of the trunk and tail. Spontaneous coiling begins at 17 hpf, peaks in frequency at ~1 Hz at 19 hpf, and then dissipates incrementally over the next 7 h (Saint-Amant and Drapeau, 1998). Recordings made from spinal motor neurons between 19 hpf and 20 hpf have uncovered two types of electrical inputs to motor neurons that are thought to mediate coiling: gap-junction mediated periodic depolarizations that trigger bursts of action potentials, and activity-dependent glycinergic synaptic bursts (Saint-Amant and Drapeau, 2000, 2001). Although glycine-mediated synaptic bursts are also depolarizing, owing to a Cl^−^ equilibrium potential ~20 mV depolarized relative to the resting membrane potential (Reynolds et al., 2008), they fail to trigger action potentials. Instead, glycinergic input is thought to contribute to the coordination of alternating spontaneous coiling, and later swimming, by preventing the bilateral activation of motor neurons through a commissural inhibitory shunting effect. Consistent with this role, *bandoneon* mutants exhibit bilateral contractions (Granato et al., 1996), however, bilateral contractions are not readily apparent until ≥24 hpf (Hirata et al., 2005). This finding suggests that activity-dependent glycinergic input between 20–23 hpf is either dispensable for the coordination of coiling, or that a transient compensatory mechanism exists. In support of a transient compensatory mechanism was the detection of maternally inherited transcripts encoding zGlyRα1, zGlyRα4b and both zGlyRß paralogs (Figure 2B). If maternally inherited zGlyRß mRNA initially compensates for the loss of embryonic zGlyRßb in *bandoneon* mutants, then translation-blocking morpholinos targeting the zGlyRß paralogs would be expected to exacerbate the onset of bilateral contractions.

In addition to a classical role in neurotransmission, glycinergic signaling in zebrafish has also been implicated in the neurogenesis of spinal interneurons. In brief, blockade of glycine-mediated membrane depolarization by strychnine (McDearmid et al., 2006; Côté and Drapeau, 2012), or by rendering GlyR activation hyperpolarizing by reversing the Cl^−^ gradient through the ectopic expression of the Cl^−^ transporter KCC2 (Reynolds et al., 2008), result in reduced neurogenesis of spinal interneurons. Additional efforts revealed that GlyR-dependent membrane depolarizations are necessary for the activation of voltage-gated L-type calcium channels, which in turn is essential for neurogenesis (Brustein et al., 2013). Thus, the inheritance of maternal RNA encoding zGlyR subunits might be essential for an early period GlyR-dependent neurogenesis, as well as in the shaping of an early behavior through synaptic transmission.

### Functional Aspects of zGlyR Subunits and Receptors

Although the initially recovered signal peptide sequence of zGlyRα4a was found to be incapable of producing functional receptors (Imboden et al., 2001a), here, we report that an alternate variant of zGlyRα4a containing a different signal peptide sequence capable of compensating for the loss of zGlyR expression in larvae (Hirata et al., 2013), yielded functional homomeric receptors (Figure 4F) with properties similar to mouse GlyRα4 (Harvey et al., 2000). Therefore, all seven zGlyR subunits are capable of contributing to glycinergic neurotransmission in zebrafish.

We also noted a difference in zGlyRα4a when co-expressed with zGlyRßa vs. zGlyRßb. In both instances, co-expression of zGlyRα4a with either zGlyRß paralog resulted in heteromeric receptors with a decreased sensitivity to glycine relative to homomeric receptors (Table 2). However, whereas zGlyRßa resulted in heteromeric receptors with a homomeric-like sensitivity to the competitive antagonist strychnine, heteromeric receptors comprised of zGlyRα4a and zGlyRßb exhibited increased sensitivity to strychnine. This phenomenon was not exclusive to zGlyRα4a as both zGlyRα4b and zGlyRα2 exhibited a similar pattern of decreased sensitivity to glycine when co-expressed with either zGlyRß paralog, but increased sensitivity to strychnine when co-expressed with zGlyRßb. Curiously this pattern was reversed with zGlyRα1, with heteromeric receptors comprised of zGlyRßa exhibiting increased sensitivity to strychnine relative to zGlyRα1/ßb receptors. As no discernable pattern exist, these findings likely represent the differential interactions of variant amino acids between zGlyRßa and zGlyRßb that lie within or near the binding site, coupled with sequence variations between zGlyRα subunits.

It is worth noting that a similar effect of lowered glycine sensitivity in heteromeric receptors relative to homomeric receptors has been observed for hGlyRα1 and hGlyRß when co-expressed in *Xenopus* oocytes (Kuhse et al., 1993; Langosch et al., 1994; Grudzinska et al., 2005). In contrast, recordings from HEK cells co-expressing hGlyRß and either hGlyRα1, α2 or α3 have reported the formation heteromeric receptors with glycine sensitivities similar to homomeric receptors (Pribilla et al., 1992; Shan et al., 2001). Therefore, some of the differences between the receptors detailed here might represent phenomenon arising from the heterologous expression of GlyRs in oocytes.

### Stoichiometry of Heteromeric zGlyRs

Experimental evidence concerning the subunit stoichiometry of heteromeric GlyRs is plentiful, but unfortunately conflicting. For instance, the first report assessing subunit stoichiometry using biochemical techniques suggested a stoichiometry most consistent with 3α:2ß (Langosch et al., 1988). In support of this stoichiometry, subsequent investigations employing substitutions of presumptive pore-lining residues found that alterations in GlyRα subunits more drastically influenced heteromeric receptor characteristics than analogous alterations in GlyRß subunits (Burzomato et al., 2003). Furthermore, results obtained using α/ß chimeras (Kuhse et al., 1993), and single-molecule imaging coupled with step-wise photobleaching (Durisic et al., 2012), lent further support to an invariant stoichiometry of 3α:2ß. However, parallel experiments employing atomic force microscopy measurements (Yang et al., 2012), a concatenated hGlyRα1-ß construct and radiometric-based metabolic labeling of monomeric subunits (Grudzinska et al., 2005), have instead suggested an invariant stoichiometry of 2α:3ß. While our results favor a subunit stoichiometry of 3α:2ß (Figure 8), we failed to explore other combinations (i.e., 4α:1ß and 1α:4ß), and therefore whether the stoichiometry of heteromeric zGlyRs is also invariant is currently unresolved. However, we can report that an apparent propensity for the formation of heteromeric receptors over homomeric receptors exists given that oocytes co-injected with α and ß cRNAs at ratios of 1:1 and 1:4 yielded glycine-evoked currents with identical EC50s and peak current amplitudes (Table 2 vs. Figure 7B). Future experiments will seek to determine whether zGlyRs also exhibit an invariant stoichiometry through the use zGlyRα1-α1 and zGlyRßb-ßb concatemers.

### *Bandoneon* Mutants

Given the role of ß subunits in the targeting of GlyRs to synapses, it is not surprising that mutations in zGlyRßb were recovered in mutagenesis screens. Of these mutants, the only viable allele arises from a premature truncation of zGlyRßb prior to the gephyrin-binding motif and the fourth transmembrane domain. Despite lacking the last transmembrane domain, functional zGlyRα1/ßb^K343X^ receptors indistinguishable from wild-type zGlyRα1/ßb receptors were formed (Figure 7B). While this finding might seem to account for the viability of homozygous zGlyRßb^K343X^ mutant fish, truncation of zGlyRßb prior to the gephyrin-binding motif predicts that zGlyRα/ßb^K343X^ receptors would be absent from synapses *in vivo*. However, the viability of this allele suggests that glycinergic transmission is present, albeit likely reduced. If true, then another process independent of the zGlyRßb subunit’s gephyrin-binding motif can facilitate the synaptic targeting of heteromeric GlyRs. Electrophysiological recordings from homozygous zGlyRßb^K343X^ mutant neurons, or the identification of synaptically localized zGlyRs via immunohistochemical labeling could provide evidence for the existence of an additional targeting mechanism.

In contrast to zGlyRßb^K343X^, the three lethal missense mutations were found to cause elevated EC_50s_ for glycine that ranged from 15% (zGlyRßb^Y79D^) to 38% (zGlyRßb^R275H^). Of these, the decrease in the sensitivity of zGlyRα1/ßb^Y79D^ receptors to glycine can be accounted for by the substitution of a negatively charged amino acid for a hydrophobic aromatic amino acid near the negative portion of the inter-subunit binding site for glycine (Figure 1C). By comparison the zGlyRßb^L255R^ and zGlyRßb^R275H^ mutations are located in M1 and at the cytoplasmic mouth of the M2 pore, and thereby distal to the ligand-binding domain for glycine (Figure 7A). Although the zGlyRßb^R275H^ mutation might have been predicted to disrupt the conductance of Cl^−^ ions given that the conserved substitution of an arginine for a histidine introduces an aromatic ring at the mouth of the pore. However, both mutations yield normal amplitudes of glycine-evoked currents (Figure 7B), suggesting that Cl^−^ conductance through the receptors is largely undisturbed. Taken together, these findings suggest that both mutations affect glycine’s ability to gate the channel in a manner similar to the hGlyRα1^G254P^ substitution which results in a ~6 fold increase in the EC_50_ for glycine (Shan et al., 2001). Lastly, it is conceivable that additional effects on receptor function not assayed here exist, such as alterations in decay time constants, or that co-assembly with other zGlyRα subunits might result in additional defects.

### Future Perspectives

The first seven *bandoneon* mutants were identified more than two decades ago (Granato et al., 1996). Since then, only one additional zGlyR subunit mutant has been isolated (Hirata et al., 2005), an eighth allele of *bandoneon*. Taken together, these mutagenesis efforts represent the screening of several thousand genomes, which although not reaching saturation, have nonetheless only uncovered mutations in zGlyRßb. It is somewhat surprising that mutations in other zGlyR subunits have not been recovered alongside the eight alleles of* bandoneon* given that all seven zGlyR subunits can contribute to functional receptors. This lack of mutations in other zGlyR subunits might represent that such mutations are lethal, or alternatively, might indicate a necessity of simultaneously disrupting several zGlyR subunits given that several zGlyR subunits exhibit overlapping expression patterns. Consistent with the latter possibility was the finding that a mutation in a RNA helicase essential for the production of mature mRNA encoding zGlyRα1, zGlyRα3 and zGlyRα4a (Hirata et al., 2013), and that the ectopic expression of dominant-negative GlyR subunits capable of disrupting zGlyRs comprised of any subunit combination, both cause motor impairment (Ganser et al., 2013; Leacock et al., 2018). Therefore, future attempts at gaining insight into the contribution of glycinergic neurotransmission in zebrafish through additional mutagenesis efforts, either forward or gene targeted using CRISPR/Cas9, might not be advisable. Instead, we propose the generation and use of a venus-tagged zGlyRα1 transgenic line bearing the R271Q mutation under the control of a UAS promoter. The venus-tagged zGlyRα1^R271Q^ line would generate dominant-negative subunits capable of disrupting both homomeric and heteromeric zGlyRs. When combined with the appropriate Gal4 line, glycinergic transmission in any neuron of choice could be silenced.

Overall, the utility of using zebrafish for investigations into the contribution of glycinergic transmission to the formation and the functional of the neural circuits that underlie behavior has been established. Hopefully the work reported here will assist others in their pursuits related to glycinergic signaling.

## Author Contributions

SL and HH designed the research and wrote the manuscript. SL and DI performed the research and analyzed the data.

## Conflict of Interest Statement

The authors declare that the research was conducted in the absence of any commercial or financial relationships that could be construed as a potential conflict of interest. The handling editor declares a past co-authorship with one of the authors that did not involve direct scientific collaboration, HH, in 2018.
