# Erratum: Characterization of the Zebrafish Glycine Receptor Family Reveals Insights Into Glycine Receptor Structure Function and Stoichiometry

**DOI:** 10.3389/fnmol.2018.00395

**Published:** 2018-10-30

**Authors:** Frontiers Production Office

**Affiliations:** Frontiers Production Office, Frontiers Media SA, Lausanne, Switzerland

**Keywords:** zebrafish, glycine receptor, picrotoxin, sensorimotor, hyperekplexia

A statement declaring a prior publication between the handling editor and one of the authors was omitted from the conflict of interest statement on the original publication.

The correct conflict of interest statement should read, in full:

The authors declare that the research was conducted in the absence of any commercial or financial relationships that could be construed as a potential conflict of interest.

The handling editor declares a past co-authorship with one of the authors that did not involve direct scientific collaboration, HH, in 2018.

The publisher apologizes for this oversight. The original article has been updated.

