# Supplementary figures and images for: Characterization of the Zebrafish Glycine Receptor Family Reveals Insights Into Glycine Receptor Structure Function and Stoichiometry
Source: Front Mol Neurosci. 2018 Sep 3;11:286. doi: 10.3389/fnmol.2018.00286 (PMC6130310; doi:10.3389/fnmol.2018.00286)

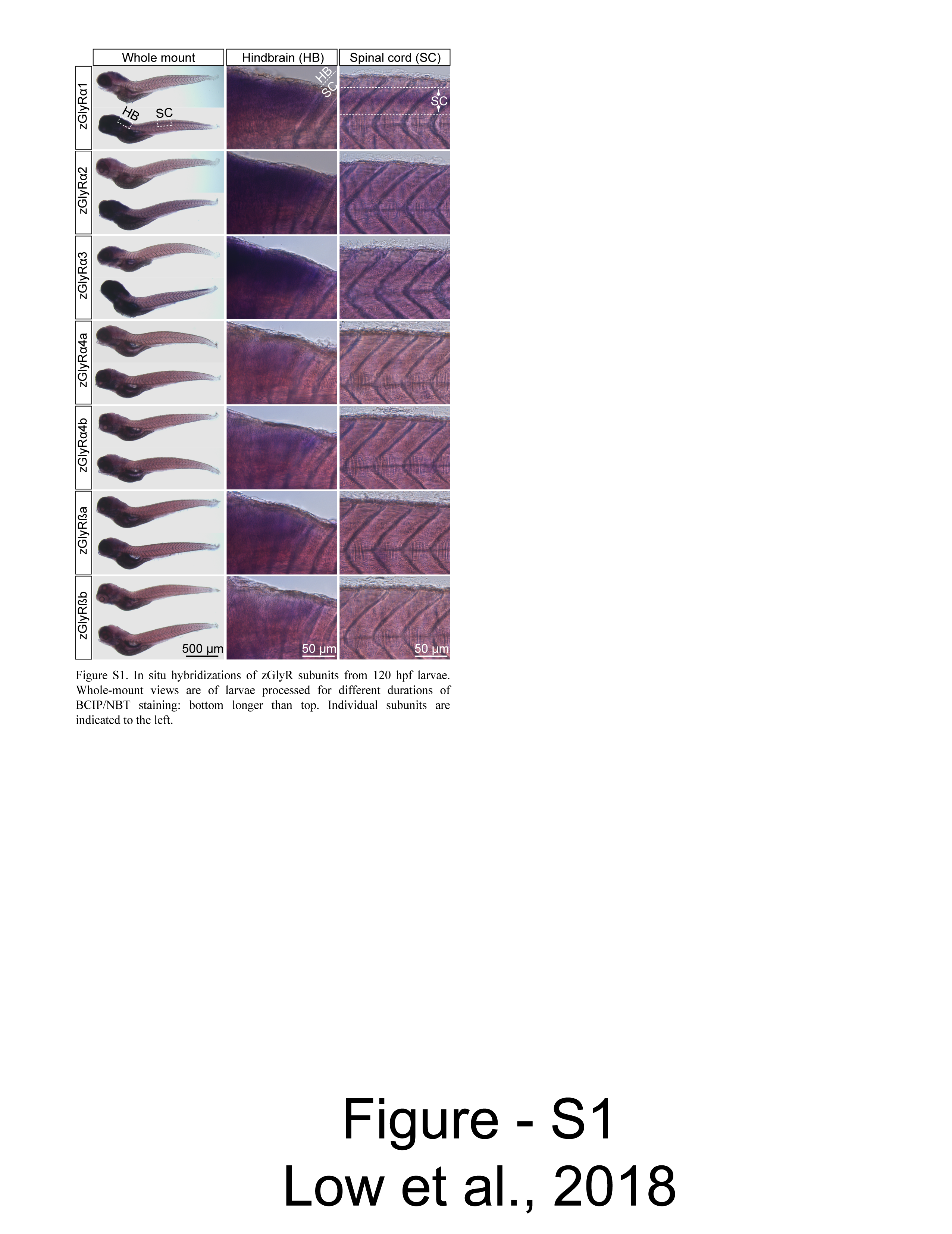

Supplement: Supplementary file 1 [file Image_1.TIF]
